# Rural Healthcare IoT Architecture Based on Low-Energy LoRa

**DOI:** 10.3390/ijerph18147660

**Published:** 2021-07-19

**Authors:** Ace Dimitrievski, Sonja Filiposka, Francisco José Melero, Eftim Zdravevski, Petre Lameski, Ivan Miguel Pires, Nuno M. Garcia, José Paulo Lousado, Vladimir Trajkovik

**Affiliations:** 1 Faculty of Computer Science and Engineering, Ss. Cyril and Methodius University, 1000 Skopje, Macedonia; sonja.filiposka@finki.ukim.mk (S.F.); eftim.zdravevski@finki.ukim.mk (E.Z.); petre.lameski@finki.ukim.mk (P.L.); 2 Technological Centre of Furniture and Wood of the Region of Murcia (CETEM), 30510 Murcia, Spain; fj.melero@cetem.es; 3 Telecommunication Networks Engineering Group, Technical University of Cartagena, 30202 Cartagena, Spain; 4 Instituto de Telecomunicações, Universidade da Beira Interior, 6200-001 Covilhã, Portugal; impires@it.ubi.pt (I.M.P.); ngarcia@di.ubi.pt (N.M.G.); 5 Computer Science Department, Polytechnic Institute of Viseu, 3504-510 Viseu, Portugal; 6 UICISA:E Research Centre, School of Health, Polytechnic Institute of Viseu, 3504-510 Viseu, Portugal; 7 CISeD Research Centre in Digital Services, Polytechnic Institute of Viseu, 3504-510 Viseu, Portugal; jlousado@estgl.ipv.pt

**Keywords:** connected health, LoRa, IoT

## Abstract

Connected health is expected to introduce an improvement in providing healthcare and doctor-patient communication while at the same time reducing cost. Connected health would introduce an even more significant gap between healthcare quality for urban areas with physical proximity and better communication to providers and the portion of rural areas with numerous connectivity issues. We identify these challenges using user scenarios and propose LoRa based architecture for addressing these challenges. We focus on the energy management of battery-powered, affordable IoT devices for long-term operation, providing important information about the care receivers’ well-being. Using an external ultra-low-power timer, we extended the battery life in the order of tens of times, compared to relying on low power modes of the microcontroller.

## 1. Introduction

Medical advances and access to healthcare available in developing countries have improved the quality of life for older adults. On the other hand, the economic growth in many developing nations has resulted in a reduction in multi-generation households [1]. In countries with fast-paced urbanization, the young people increasingly migrate for economic reasons, while the retired population remains in their rural homes during this initial migration phase. This trend has left an increasing number of old age population alone or in some areas they take the additional role for caring for their grandchildren [2]. Often this aging population can lead independent and productive lives in their advanced age, and many choose to remain in their home as long as they can [3]. Moreover, with the aging population in rural areas absent government regulations to promote rural investing, there is less incentive for technology and telecommunication providers to invest in bringing stable electricity and network connections to these areas [4].

In addition to telecommunication and electrical infrastructure, rural environments pose other challenges, including sparse population, road conditions, distance to urban areas, and lack of local skilled labor. While these aspects vary from location to location, designing such critical systems as Active Assisted Living (AAL) should holistically consider all constraints.

For the independent living of older adults in rural areas, providing cost-effective, low-maintenance solutions is essential. This paper proposes an architecture that would meet these requirements based on recent advancements in the Long Range (LoRa) network, fog computing, and low Earth orbit (LEO) satellite connectivity, using state-of-the-art energy efficiency. Such proof of concept for supporting older people using the LoRaWAN network, the Things Network, and ESP-32 microcontroller was already presented in [5] focused on providing real-time data, and in this paper, the concept is extended.

LoRa falls under the low-power wide-area network (LPWAN) type of communication technology. LoRa is a proprietary physical layer protocol that transmits in unlicensed frequency bands reserved for industrial, scientific, and medical (ISM) use. The data-link layer, most commonly used in Internet of Things (IoT) devices, is called LoRaWAN and is an open standard. LoRa and LoRaWAN only define the communication protocol and often come in a combined hardware package with microcontrollers. LoRa is designed for low-energy sleep mode, and many IoT microcontrollers also have low-energy states.

Fog computing extends the cloud services to the edge of the network. It makes computation, communication, and storage closer to edge devices and end-users, which aims to enhance low-latency, mobility, network bandwidth, security, and privacy [6]. Fog computing introduces another layer between the cloud and the end devices, called the fog layer. The end devices also referred to as IoT nodes, are located in the terminal layer. IoT nodes, by definition, must be connected to a network, and they usually include various sensors or actuators that interact with the physical world [7]. Specifically, in fog computing for medical applications, they connect to a device called e-healthcare gateway that provides services and offers cloud connectivity.

The IoT nodes are usually battery-powered. Due to multiple constraints in rural areas, it is essential to implement efficient battery-saving protocols using sleep modes when the node is idle. To accomplish long battery life ultra-low-power (ULP) modes, that are built-in with modern microcontrollers and external ULP timers are used. However, for the application to be practical, the device would need to wake up either after a specific time interval or via an external interrupt to conduct the required tasks.

## 2. User Stories

To design a more functional architecture, we identify four user stories that present different challenges in a realistic scenario when monitoring a person’s wellness.

### 2.1. Story: Finding a Lost Person

Seniors with deteriorating mental health, including dementia, can wander away from their residence and get lost. A holistic approach for identifying when the person left their residence and identify the current location is needed for timely intervention by rescue teams or search groups from other residents in the area. In a rural area, people might live alone and have infrequent visitations from other community residents, and they might not be identified as missing for many days. Chances of finding the person can be increased by equipping everyday used clothing items such as a winter jacket, outdoor shoes, a belt, or a personal item with a battery device. This device will send a signal that can be triangulated or obtain and transmit GPS coordinates. Such a signal should be received by any other healthcare gateways in the area. A healthcare gateway used by the person’s residence should have an algorithm for detecting and raising the alarm when certain events occur that indicate the house resident is expected to be at home but is not present and should be considered lost [8]. For privacy reasons, the healthcare gateway should not transmit such data if the person was away due to normal daily activities depending on the time of day and has returned within the expected time.

A wireless healthcare service system for seniors with dementia is proposed in [9]. The authors suggest using various wireless solutions and rely on the availability of a GSM network to send emergency signals. In a rural environment, a GSM network might not be available. However, any proposed system should support all necessary technology to locate the person.

### 2.2. Story: Detecting Abnormal Changes in Daily Living

Older people tend to establish daily routines that help them organize their lives and create a comfortable environment. Having repetitive days is often a good sign for the care providers. On the other hand, sudden disturbances can indicate possible underlying problems with their health or well-being. One of the most important routines for a healthy life is healthy sleep. Health issues or emotional stress can project onto a person’s sleep patterns. Care providers would benefit from a non-invasive system present in the sleeping area to monitor the person’s sleep. The monitoring will include the time of day when they go to bed and wake up, the time it takes them to reach a sleep state, and potential disturbances in their sleep. Such a system should be a part of the environment and not require direct interaction like attaching wearable sensors and prompts.

Based on previous research [8,10], we have learned that passive-infrared (PIR) sensors positioned above a person’s bed could identify these sleep parameters and also do it in a non-invasive and privacy-preserving way [11].

In cases where the health care receiver’s actions can improve the condition, a recommendation system such as the COllaborative HEalthcare SYstem model (COHESY), described in [12,13,14], can be used to recommend activities. For example, if evening walks are determined to improve sleep in a specific group of people, such activities could be automatically recommended to persons with similar health or demographic characteristics.

### 2.3. Story: Environmental Safety Monitoring

Relatives and care providers would benefit from knowing that seniors live in a safe environment, including reduced risks. While notifying relatives or care providers in case of emergencies is essential, false alarms cause other sets of challenges [15], causing a need for a balance in the accuracy and frequency of notifications. Due to the lack of infrastructure in rural areas, common defects can have severe and even dangerous consequences. Having standalone, easy to install sensors to monitor electricity and water usage, detect water leaks or fire hazards can significantly improve the inhabitants’ safety. There is great potential to equip everyday items with inexpensive sensors that will, in turn, communicate with the in-house or remote gateway. The following are some examples:

- A compressed canister with gas used for the cooking stove can be equipped with a hall sensor to detect when it is being turned on and even have a safety shutoff valve triggered by a timer if left on beyond the expected time.
- An inductive coil placed around an electrical lead wire can induce a signal for a sensor to monitor electrical usage. Multiple devices can be monitored by monitoring the consumption of common power lead.
- A water sensor can be placed on the floor to detect any water leaking in the house, either from running water or rain.
- Magnetic sensors on the doors and pressure sensors on floor mats can detect when a person enters or leaves the house.

### 2.4. Story: Medicine Dispenser

A patient with prescribed medication would benefit from having a medicine dispenser device to remind them and keep track of when the medicine needs to be taken. Depending on the type of medicine, the system would prevent doubling the dosage when the care receiver does not notice or ignores the alerts and does not take the previously dispensed dose on time. A healthcare provider would benefit from a centralized system that would track compliance with the prescribed treatment. The doctor would use the same system to adjust the prescribed frequency of the medicine already in the dispenser. Thus, it is required for the dispenser to be connected to the system via the healthcare gateway. The pharmacy and the distributor would have a complete inventory. They will refill all the medicine dispensers in the area with minimal trips while ensuring that all medicine will be taken before the expiry date.

## 3. Architectural Challenges

With the push to increase connectivity in developing and under-developed countries, regions with a dense population such as cities, towns, and suburban areas are increasingly interconnecting. This trend would accelerate soon, especially with the push towards 5G networks. On the other hand, rural areas are shrinking in size, and the average age of the population is increasing [16], thus providing connectivity is not a priority for network providers. Solutions such as mobile and satellite internet, for direct connectivity to the cloud, are prohibitively expensive. Furthermore, by the nature of rural areas and farmlands, the houses are sparsely positioned, cable and Wi-Fi links are technologically not viable due to signal deterioration. Thus, we identify the following constraints that the architecture must meet:

- Internet connectivity is of low quality, expensive, or nonexistent.
- Houses are mostly spaced out at a distance of at least 100 m.
- Electricity is not reliable, and blackouts are to be expected.
- The care receivers are unable to troubleshoot or repair IoT devices.
- Servicing can be done from once in few months to once in a few years.
- The systems must be affordable, even for a few participants, and scaling up reduces cost.
- When storage is limited, the most valuable data has a higher priority over the most recent data.

The lack of persistent Internet connectivity implies a self-sustained network that relies on one or few links for connecting to the cloud. The limitation for Internet connectivity requires an architecture that would support local processing as the data cannot be fully or immediately uploaded to the cloud. The sparse distribution of the houses and the need to minimize cost require long-range wireless network links.

The lack of reliability of electrical power is one factor that would require a battery backup for the devices. However, difficulties and expenses related to the installation of powered devices, especially in older houses that might not have electrical installations in all rooms, increase the preference for battery-powered devices. The battery capacity should be maximized so that the active time of the devices is sufficiently longer than the maintenance window when the operator could service or replace batteries. To extend the lifetime of the battery-powered devices, it is crucial to have low power and sleep states such that the device would minimize the time needed to remain in a higher power consummation state.

### 3.1. Communication Synchronization

A big challenge for devices that remain in sleep states for prolonged periods of time is communication synchronization. The energy constraints prohibit a device from having a complete picture of the state of the environment, the network, and other devices.

Communication in the network is inherently sparse based on the assumption that energy is not reliable. Each IoT node and healthcare gateway should have a built-in real-time clock and calendar chip and a battery-powered circuit. If power is lost due to power grid blackout or bad conditions for renewable energy harvest, a timer-based battery switch starts the gateways at the predetermined communication time slot. A real-time clock or internal timer will also wake up IoT devices with buffered data to transmit to the network.

### 3.2. Energy Conservation

In multiple papers, energy conservation in LoRa networks has been studied, most of which address the LoRa and LoRaWAN parameters and the tradeoff between energy consumption, reliability, and distance [17,18,19,20]. Minimizing data transmission is a practical energy-saving approach, especially in our proposed architecture that relies on slow data links. Techniques to achieve this includes compression and data pre-processing, and filtering. This paper proposes using external ULP timers to replace the modern microcontrollers’ built-in ULP modes, as presented in Section 4.7. This approach enables nano-Ampere sleep currents for the IoT device.

Energy efficiency in LoRa based networks, especially when using the LoRaWAN protocol, is a well-researched topic. An energy consumption model based on LoRa and LoRaWAN is described in [17]. The authors study the impact of LoRaWAN parameters such as acknowledged transmission, spreading factor, coding rate, payload size, and communication range on the sensor node consumption. Their model is based on LoRaWAN Class A. The optimization study showed a tradeoff between the LoRaWAN communication range, the spreading factor, and the transmission power. This optimization study is very interesting to choose and configure LoRa/LoRaWAN parameters [17]. Energy efficiency optimization of LoRaWAN using an open-source simulator is presented in [18].

Optimization strategy in LoRa networks can be done by transmission parameter selection. There are 7620 possible parameter combinations [19]. It is possible to use different network parameters, which result in acceptable link quality but with energy consumption that differs 100 times [19]. The LoRaWAN specification does not provide configuration selection mechanisms; thus, most implementations use static parameters. For high communication ranges (greater than 10 km), the transmission power must be fixed to 20 dBm with Spreading Factor (SF) set to 12 [17].

Energy efficiency for multihop LoRa networks was studied in [20]. The authors used an adaptation of the TSCH MAC protocol for a long-range operation which addresses most of the challenges created in multihop LoRa communications. A time division mechanism synchronizes nodes’ wake-ups for constrained nodes. They also implement a channel hopping mechanism to diversify transmissions over different channels in a power-efficient manner.

## 4. System Architecture

**Figure 1:**
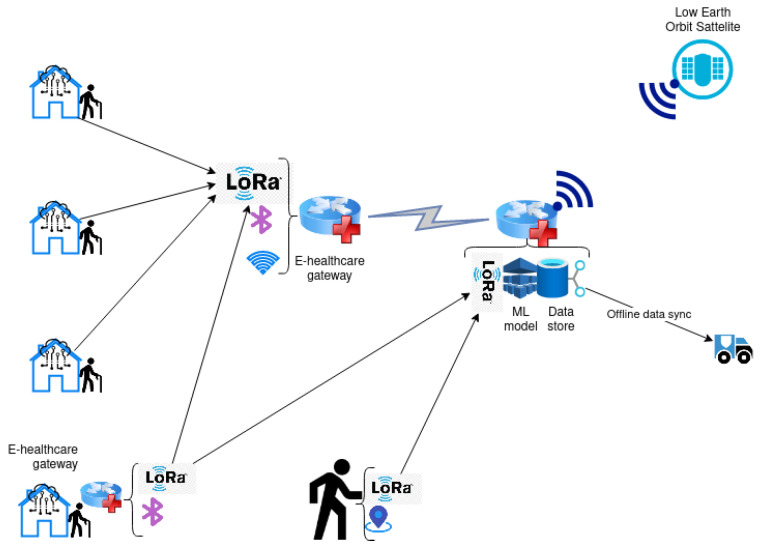
System architecture for rural area network for healthcare IoT.

**Figure 2:**
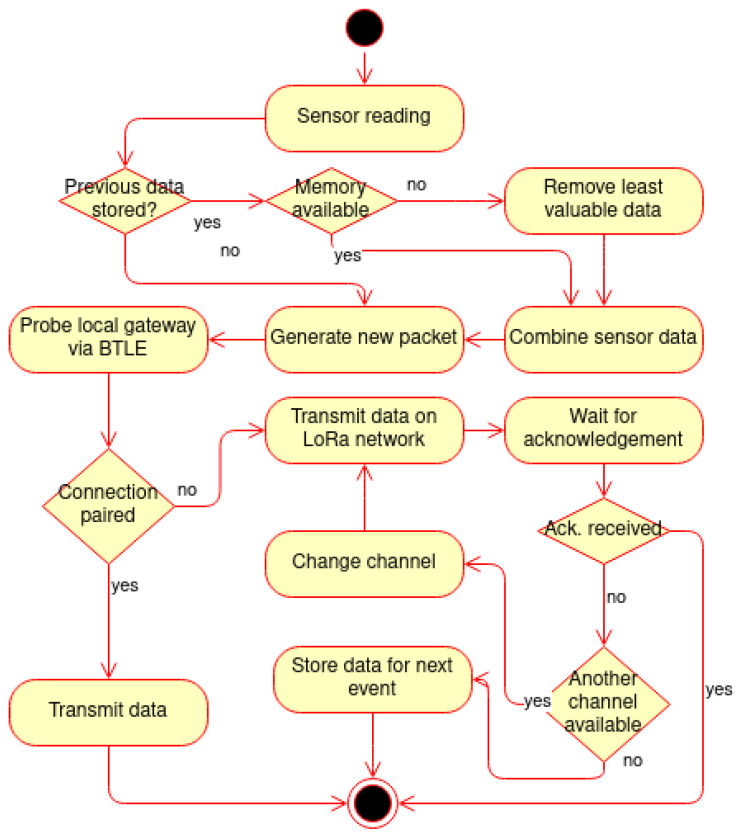
Data transmission decision process workflow.

To address the lack of internet connectivity and the distance between connected devices, and the inability to establish an Ethernet connection, we propose a flexible fog network architecture where the primary communication channel for long-distance traffic is based on LoRaWAN. In Figure 1, we present a sample connectivity scheme. IoT devices in a home, including sensors, can have two modes of connectivity. They could either be connected to an in-house healthcare gateway or have embedded LoRa transceiver radio and communicate to the neighboring gateway. Wearable devices with LoRa radio transmit can communicate with any healthcare gateway in the network. The process for sending data is shown in Figure 2.

Unreliable electricity and difficulty to set up charging outlets and the risk of them being unplugged by the house occupants is addressed by relying only on battery-powered IoT devices. The healthcare gateways also require reliable battery operations with recharging the batteries either via the power grid when electricity is available or using renewable energy sources such as solar panels. The need for battery operation and the long servicing time poses energy conservation as the primary challenge in our design, and we address this in the following sections.

To ensure cost efficiency from small implementation to scaling up, we propose inexpensive off-the-shelf components integrated into sensor enclosures for small quantity deployments. Then, when a need arises for larger deployments, some components can be reduced or combined to save material costs. An example of such cost saving through consolidation is creating custom boards with multiple sensors used by an IoT device, built-in sleep timers and clock chips, and a single power supply circuit.

The proposed architecture has several built-in constraints resulting from batteries as the primary energy source and LoRa as the dominant protocol for distant communication. LoRa is designed to be energy efficient; however, that comes at the cost of very low bandwidth. With the requirement of a long-lasting battery, energy conservation is the primary challenge. Ensuring maximal energy conservation is accomplished by reducing higher power consumption events and extending sleep states’ ability. IoT devices should be awake only long enough to collect and transmit data. The challenges introduced by power cycling devices and long sleep periods are the following:

- No internal real-time clock/calendar for time-stamping events.
- Communication is unreliable, and data transmission is not always possible.

### 4.1. LoRa and LoRaWAN

LoRa is a proprietary radio modulation technology licensed by Semtech Corporation. LoRa uses unlicensed ISM bands: 868 MHz in Europe, 915 MHz in North America, and 433 MHz in Asia [5]. The Bidirectional communication is done using Chirp Spread Spectrum (CSS) modulation, or as defined by [21] a Frequency Shift Chirp Modulation (FSCM), where each symbol is sent using a wide frequency band. The advertised communication range of LoRa is more than 15 km for suburban environments [22]. The long-range and low-power nature of LoRa makes it a likely candidate for smart sensing technology for health monitoring and environment monitoring [23].

There are three classes of end-devices (Class A, Class B, and Class C) which differ in the timings of the receive windows [24]. Class A has receiving windows after the send windows. Class B has received windows at timed intervals. Class C has continuous listening.

There are five configuration parameters for LoRa radio: Transmission Power (TP), Carrier Frequency (CF), SF, Bandwidth (BW) and Coding Rate (CR) [22]. The rate at which the data is transmitted depends on three physical layers parameters: SF, BW, CR [24]. SF indicated how the signal is distributed in time. The more time it takes to send the packet, the lower the minimum RSSI. A higher coding rate increases redundant bits, thus making the transmission more reliable. However, it increases time and energy consumption. A higher bandwidth reduces the transmission time but at the cost of lowering the noise floor.

On top of the physical layer, the MAC layer protocol defines the topology of the network. An analysis for the creation of ad-hoc MAC layers is presented in [25]. A synchronous-based, medium access control (MAC) protocol, MAC on Time (MoT) was studied with the potential to have reduced energy footprint [26]. An alternative MAC layer protocol named LoRaBlink was discussed in [27]. However, in this paper, we review only LoRaWAN, which uses a star topology and is most often used in LoRa deployments. The LoRa modulation is proprietary; however, the LoRaWAN is an open standard being developed by the LoRa Alliance. It is designed mainly for sensor networks, where sensors send data to the server infrequently (one transmission per hour or even days) [23]. LoRaWAN is based on the ALOHA protocol. The end devices communicate directly to an always-on gateway when they have data ready to send [28]. The downsides of this star topology include: cost of the base station(s); the need for more frequent battery replacement for remote nodes; and need for high SF and a higher chance of collisions for remote nodes [20].

The analysis conducted in [29] shows that the long-range transmissions of LoRa are vulnerable to multiple security attacks. The possible attacks include compromising nodes to obtain network keys, jamming, replay, and wormhole attacks. The security measures in the LoRaWAN protocol are left to be implemented by developers and manufacturers, thus providing room for bad implementation in some devices to introduce risk for the network.

### 4.2. Fog Computing and E-Healthcare Gateway

With the evolution of healthcare, Fog Computing is one of the possible solutions for the improvement of healthcare treatments in rural environments for the performance of all treatments remotely [30]. Fog computing is a paradigm that addresses connectivity constraints. Our architecture focuses on moving computation closer to the edge. All IoT devices have sensors and microcontrollers to conduct data processing and only transmit the minimal amount of data needed to reach the cloud. The primary constraint in the proposed fog topology is the limited bandwidth of the LoRa network. The gateway has bi-directional communication with the IoT devices and the communication hub for the area, typically a village. Communication with nearby devices located in the same house can be established with other protocols such as ZigBee [31], or Bluetooth 5, which can have up to 200 m range [32].

In the proposed architecture, the healthcare gateway collects raw medical signals from sensors, conducts data pre-processing, and feature extraction [33]. Depending on the type of data, the system could utilize different Machine Learning (ML) models to extract meaningful information from the raw data. When ML is used for decision-making, the gateway determines actions based on the most recent ML model. Such decisions considering the identified user stories and include determining alarm activation for a missing person or abnormal changes in daily activities, environmental controls, and health monitoring.

The main constraints for applying ML in the edge nodes are related to the limited computing and battery power. Therefore, the ML model training is conducted in the cloud, and the model is deployed on the e-healthcare gateway. The fog network is used to transmit up-to-date models from the cloud to the e-healthcare gateways [34]. When selecting the algorithm, considerations should be made that updates are done via low bandwidth links and that the data has a low sample rate due to energy constraints. In addition, the pre-trained ML models that would be used on the edge nodes should be as lightweight as possible, for example, by using data structures with lower precision and, consequently, lower memory footprint. The specific selection of the ML algorithm and implementation details would depend on the use case, the sensor types, and the data quantity. However, the proposed architecture would apply to any algorithm that could meet the said constraints.

**Figure 3:**
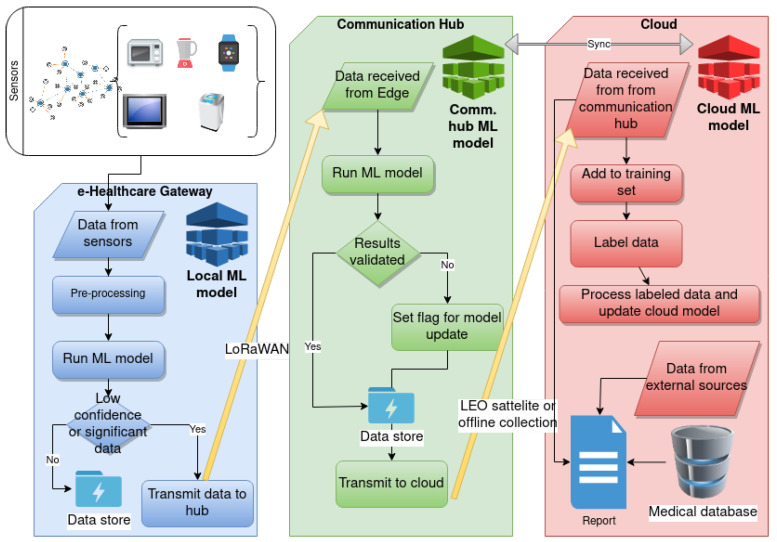
Machine learning workflow using Fog based architecture.

In Figure 3, we present the workflow for data transmission and data processing in the proposed architecture. The IoT nodes pass the data to the e-healthcare gateway, shown in the blue/left block, where the local model is run. If a significant event is detected, a communication channel is established with the communication hub, which can communicate to the cloud or care providers if the alert is triggered. Additionally, in cases of low confidence classification or when ambiguous classification occurs, the data is sent for further processing and labeling in the cloud. This means that all outliers and classifications made by the lightweight ML models on the edge nodes would eventually be validated by more performant ML models on the cloud side. On the communication hub, shown in green/middle block, the results are validated, and if a significant discrepancy is found, the hub will set a flag to download the latest cloud model and update edge ML models. All data received by the communication hub is stored and transmitted to the cloud either by network link, such as LEO satellite, or via offline data collection during the service period. The cloud, shown in red/right block, is responsible for generating the ML model and providing an interface for labeling data. In addition, the cloud gathers data from external sources such as medical databases and patient records. Therefore, all services offered to care providers are centralized in the cloud.

To illustrate the ML process within the proposed architecture and to state the challenges given the architecture constraints, we will use a scenario that falls under the user story “Detecting Abnormal Changes in Daily Living”, described in Section 2.2. More precisely, we consider how sleep monitoring of a person can be used to indicate underlying health conditions. In our previous work, we have elaborated that a combination of piezoelectric and PIR sensors that measure different physical properties, thus minimizing the possibility of noise signal contamination, show a strong correlation, and can be used to detect the person’s movement in bed during sleep [35]. For the data collection, the microcontroller can operate in the ESP32 Deep Sleep state. This sleep pattern of the microcontroller is known as ULP sensor-monitored pattern and the stated current usage is 10 μA [36], while higher when it is part of a microcontroller. Additionally, the sensors should be modified to minimize elements that dissipate energy in the form of heat, such as the linear voltage regulators.

The data collected from the sensors is stored in the real time clock (RTC) memory, which, based on the hardware revision, is 8MB or 16MB. When the RTC memory is full, the microcontroller wakes up, compresses the data, and begins transmission to the e-healthcare gateway. The e-healthcare gateway must first filter the data to determine its validity and filter for possible noise by correlating data from multiple sensors. Then, using its pretrained ML model, the gateway detects events of interest about the care receiver’s sleep. The challenge presented in this case is transmitting the sensor data and labeling it for reinforcement learning in the cloud. The LoRaWAN bandwidth limitation implies that not all data can be continuously transmitted to the communication hub and from there to the cloud. Thus, the system should contain thresholds either by the number of non-zero readings from the sensor or by the number of low confidence classifications. The data can then be analyzed by a medical specialist who can label the data by the patient’s current diagnosis. This may include if the patient is known to have developed respiratory symptoms or has been diagnosed with sleep apnea. More granular labeling can be made if the care receiver, in addition to non-invasive sensors, was using body area sensors such as a wristband to detect pulse and oxygen saturation. Thus, data from patients that use such sensors should be prioritized for transmission and, upon reaching the cloud, is added to the reinforcement training set.

To minimize the energy dissipation of dropped packages from the IoT sensor nodes, the gateway should maximize the receive slots to capture data from the nodes. Furthermore, sleep times for the healthcare gateway should overlap with all potential sending nodes’ sleep times. However, the dependency of the IoT cloud analytics or storage facility must be minimized for the exploitation of the proposed solution [37].

**Figure 4:**
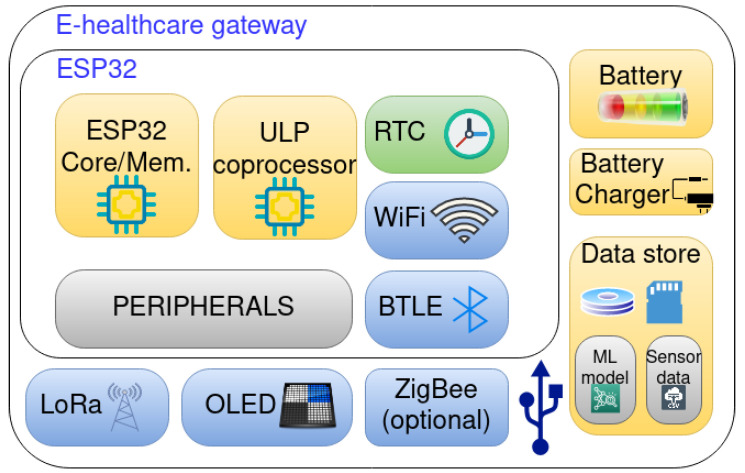
E-healthcare gateway components.

For ease of operation and maintenance, the gateway will feature functionality for auto-update, and remote reboot [38]. A block diagram of the e-healthcare gateway is shown in Figure 4.

The healthcare gateway should manage user interfacing with the system either as a built-in interface or via a separate user interface (UI) module. Smartphone interface could be used as an extension but should not be mandated as it will introduce additional complexity. A consideration for accessibility should be part of the UI design.

### 4.3. IoT Devices

**Figure 5:**
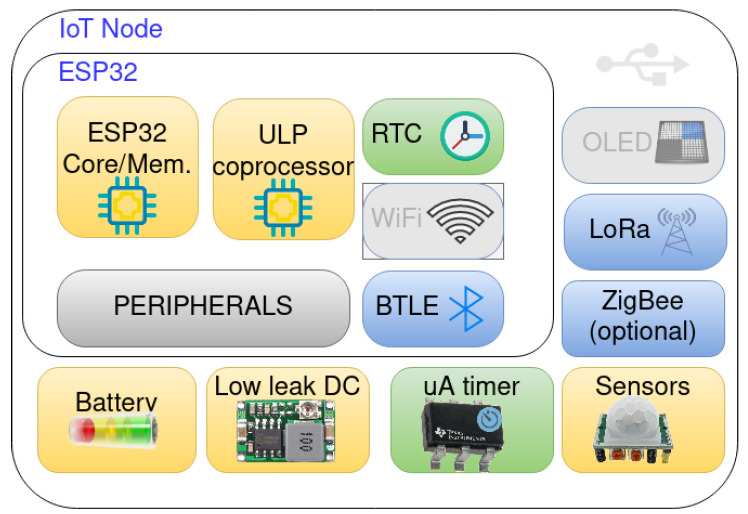
IoT node components.

In the proposed architecture, the IoT devices collect data. In the topological sense, they are part of the fog network as they connect to the healthcare gateways through a network link. These nodes consist of a microcontroller, optional communication chips and timers, and sensors. A diagram of the IoT node components is presented in Figure 5. The sensors are part of the device’s enclosure, or in some cases, are connected by wire, depending on the type of measurement conducted. To reduce cost, the number of sensors on a node is maximized. However, each sensor could independently be turned on or off to facilitate energy-saving schemes. While data processing is primarily done on the gateways, the IoT nodes also reprocess data. Decision-making for simple logic that can be programmed on the microcontroller is also done directly on the node. For example, in the environmental safety user story, a water shutoff is automatically initiated by a node that monitors water usage if certain limits are reached. Such actions should always be reported to the healthcare gateways, with the possibility to receive overrides. The UI on IoT nodes should be limited to only servicing interfaces and alerting the care receivers, such as with the medicine dispenser.

IoT devices in healthcare might not have predetermined patterns for energy usage, especially when they measure events related to the person who receives the care. For example, devices that measure sleep-related parameters will consume energy proportional to the number and duration of monitored activities. The same applies to other aspects of activities of daily living (ADL). Thus, different behavior in ADL could lead to a different energy profile of the devices. Proper battery management is needed to estimate and adjust the activity periods to extend the battery life to the expected period. A battery level measurement could be conducted to receive feedback of battery consumption, and battery usage could be estimated from the duration and energy profile of different stages of the IoT device, such as boot-up, data collection, data processing, and communication. The energy consumption should be measured for each IoT device and a detailed energy profile created. This energy profile will then be stored in the persistent memory of the device itself. The battery capacity profile should also be established [39]. Different device profiles should be created depending on the speed of battery depletion to compensate and extend the battery life. Techniques must be customized depending on the device function but could reduce sensor sampling frequency, reduce communication windows, and increase the threshold for data collection. For example, the ML models such as decision trees should be pre-programmed or received from the network. They should be structured to use minimal computing and battery power, for example, by using data structures with lower precision and, consequently, lower memory implications.

### 4.4. Rural Healthcare Data and Communication Hub

The fog layer needs to be connected to the Internet (TCP/IP network) to allow access to the cloud services, including data storage and analysis. Due to network delays and bandwidth limitations, a simple internet connectivity link is insufficient as any down-times would introduce congestion if the data is buffered on the e-healthcare gateways. Thus, we propose a centralized communication hub that can serve one or several adjacent rural communities depending on geography. This communication hub would have data storage and additional data processing capabilities. Such a centralized hub could benefit from having more expensive technologies to implement on the e-healthcare gateways, such as satellite telephones for emergency communication, dedicated microwave links, and high gain antennas for GSM where a signal too weak to be picked up by mobile phones exists.

The connectivity must be made by one of the available options (cable, microwave, 3G/4G) or by another network access technology that may become available in the future, namely 5G, TVWS or LEO Satellites. In addition, several devices available on the market can work as a Gateway, with WIFI or 3G/4G connection. For example, the Dragino Gateway LG308, an open-source source LoRaWAN Pico Gateway allows bridging LoRa wireless network to an IP network via WiFi, Ethernet, Or 3G/4G cellular via optional Long Term Evolution (LTE) module that results from an application of the WiMAX (Worldwide Interoperability for Microwave Access) spectrum [40].

With the emergence of 5G mobile telecommunications, we will see the development of devices supporting this technology, allowing a range of differentiated and possibly low-cost use, combined with a very efficient coverage. However, we will always be conditioned to mobile network coverage, which will not always be economically viable in remote and rural regions, making this not a technology of the first choice in some contexts, like other mobile communications based on 2G, 3G, and 4G.

The future will involve using the Internet supported by LEO satellites, which is increasingly close to reality. The mass delivery of LEO satellites is intended to provide coverage of high-speed Internet services to all regions of the world [41]. Recently, major technology companies such as Google and SpaceX have entered into agreements to provide data, cloud services, and applications to domestic and business customers all over the world soon. Likewise, it was reported that SpaceX will also install ground stations within Google’s data centers that will connect to Starlink satellites, allowing Internet services through the Google Cloud platform. Starlink’s satellites are over 60 times closer to earth than geostationary satellites, resulting in lower latency and the ability to support Internet services typically not possible with traditional satellite [42]. This will undoubtedly be an unprecedented revolution that will democratize access to the Internet anywhere in the world.

### 4.5. Bandwidth Limitations

A requirement for remote devices that would have to operate during long periods between servicing is receiving remote updates, including software updates and updates to ML models. The LoRa alliance provides a specification for remote firmware updates called Firmware Updates Over the Air (FUOTA) [43]. However, updating the firmware over the air is complex, difficult to execute, and requires a series of technical requirements. It usually involves several steps to ensure that the update is successful, namely:

1. Identify the devices that need to be updated and place them in multi-cast groups;
2. Generate a binary firmware update file specific to the platform of the device to be updated;
3. Sign the binary file with a private key to ensure the integrity and authenticity of the update file;
4. Send the binary file to the device group to be updated;
5. Each device will have to verify the signature to authenticate the file as well as the authenticity of the origin;
6. Each device will need to install the firmware update;
7. Each device must report the status of the update operation (success or failure).

This information is collected by an integrated device management platform that monitors its status. Some of these steps (2, 3, 5, and 6) are very specific, depending on the devices, mainly conditioned by the following characteristics:

- Device microcontroller architecture (ARM, X86, etc.);
- Device memory capacity;
- Whether the device uses an operating system;
- Whether the device uses a secure boot-loader;
- Whether the device’s firmware is designed for partial updates;
- Whether the device has a cryptographic hardware accelerator;

These restrictions make it impossible to standardize a FUOTA process that works for all devices on all LoRaWAN networks, in addition to the low bit rate (50 Kbps) intrinsic to LoRaWAN networks [44]. To address this issue, the LoRa Alliance, the industry body behind the LoRaWAN standard, created the FUOTA working group to define the basic needs to enable an efficient FUOTA over LoRaWAN. This has resulted in new specifications to cover multi-cast, fragmentation, and time synchronization topics, which are essential resources for an efficient FUOTA. In [45], the authors describe these new LoRaWAN specifications and examine how the new features can enable a fast and efficient firmware update, using a simulator developed for this purpose.

The use of higher bandwidth networks such as WiMAX (802.16), wireless communication network, currently using microwaves with frequencies between 2 and 11 GHz (the initial version used frequencies between 10 and 66 GHz) with communication rate up to 70 Mb/s and range up to 50 km, is an alternative to establish the connection between LoRa Gateways and the Internet, in areas of difficult access, such as remote, mountainous and rural regions where fiber and wireless communications cable are not possible to install. Although very promising, this technology ended up being overshadowed by 4G in mobile communications, particularly in the use of LTE, which took away market space for WiMAX. However, the latest version of WiMAX is a strong bet for wireless communications, working at rates of 1 Gb/s. However, its use is severely restricted, being used in particular situations [46].

Another viable alternative to allowing data communication between the e-healthcare gateways and communication hub is the TVWS technology, as presented in [5], which is inspired by WiFi networks and is considered the most robust and economical solution in rural environment scenarios. With the transition from analog TV to digital transmission, the use of TVWS, which are the underutilized portions of terrestrial TV bands, for data communication purposes is becoming increasingly evident worldwide. It uses the spaces between television channels that are not used by the frequency range of television broadcasts (between 300 MHz and 3 GHz). These frequencies can be used to communicate over long distances (up to 200 km). Several pilot projects have already been successfully tested. The essence of the unlicensed spectrum is that any certified device can operate within it, with only minimal restrictions on the uses that can be made. The unlicensed spectrum encourages manufacturers to collaborate in the development of open standards and compete to deliver low-cost components and user equipment. However, there are limitations in the use of this spectrum, namely due to the difficulty in finding a common universal standard [47]. Having an updatable database online on the routers is the main obstacle since each country has its free frequencies. Within each country, the frequency ranges available may vary from region to region, which significantly complicates implementing a standard system using TVWS. However, it is still a good alternative for wireless long-distance communications as its broadcast nature could allow for transmitting software updates and ML models to the entire network.

More recently, a new model for wireless communications has emerged that appears to complement existing technologies to solve the limitation of coverage of the terrestrial network. This satellite network has been considered in the construction of IoT systems to provide global and uninterrupted IoT services. Considering the limited energy consumption of the IoT device, the LEO satellite is a good candidate for providing IoT services due to its low propagation loss. Meanwhile, the LEO satellite also has advantages in the low propagation delay and in provisioning continuous global coverage, which satisfies the requirements of a global IoT network. The LEO satellite generally acts as an access point in a satellite communication network, making a network like LoRaWAN in the topology domain. Therefore, when combining LoRaWAN with the LEO satellite system, LEO satellites mainly play the role of LoRaWAN gateway, which is used to acquire data from devices, operate and control access [41].

In order to meet the needs of IoT by satellite systems, in [48] the authors introduced LEO satellite constellation technology to support IoT telecommunications systems. These have unique advantages compared to geostationary satellite communication systems since they orbit closer to the earth. The constellation of LEO satellites (usually less than 2000 km) is more efficient in terms of time. In terms of propagation delay, quantified by a round trip time (RTT), the LEO satellite constellation has an RTT of less than 100 ms. In comparison, the RTT of geostationary systems is greater than 600 ms. Thus, the constellations of LEO satellites are presented as a good bet for the future to make Internet access available in remote regions, rural areas, among others, where terrestrial technologies, by cable or fiber, are not possible to be installed.

### 4.6. Memory Persistence in Sleep Modes

As transmitting data via LoRaWAN has energy implications, an application that aims to reduce consumption could buffer the data gathered by the sensors and transmit more infrequently depending on the data size and the LoRaWAN packet size.

Let $p_{l}$ be the power used for sending the LoRaWAN packet and $t_{l}$ be the time needed to send the packet. Let $n_{p}$ be the byte-size of the packet and $n_{s}$ be the sample size of data collected in one sampling interval. Let $p_{s}$ is the power consumed during sleep. We will assume that this value is much lower than $p_{l}$ and would not consider it. The energy savings when using data buffers is *E*, shown in Equation (1).

(1) $$E=\left(\left\lfloor \frac{n_{p}}{n_{s}}\right\rfloor -1\right)\;*\;p_{l}t_{l}\;(\mathrm{if}\;p_{s}\ll p_{l})$$

**Figure 6:**
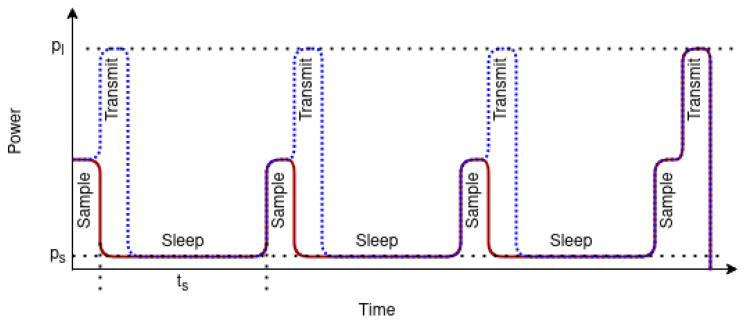
Energy savings by combining data in a single data frame.

It is illustrated in Figure 6, where the blue/dotted line indicates a normal scenario where data is sent after each sample and the red/solid line is the energy saving mode where a message is composed of multiple data readings to form a single data frame. $\left\lfloor \frac{n_{p}}{n_{s}}\right\rfloor$ is equal to four. This indicates that the data from each sensor sample is less than or equal to 1/4 of the message size. The ‘−1’ in the Equation indicated that we could not avoid the final transmission once the packet size is reached. In the case of a noisy channel with a high probability of collisions, this approach further reduces the probability of collision by reducing the number of transmissions.

Another option is using persistent memory, such as a micro SD card, to preserve the data. In this use case, additional energy is used to read and write data on the SD card each time the sensor reading is performed. We must read data every time the sensor measurement is done to determine if the buffer is full and ready to transmit. Let energy used for the write and read data step $E_{rw}$. Since the data is written to permanent memory, the microcontroller could be placed in a power-off state to be awaken by external ULP timer. If we consider that the external interrupt uses power much lower than the microcontroller ($p_{ext}\ll p_{s}$), in the sleep state, the energy is saved for the entire sleep interval $t_{s}$. The average power used during boot-up is $p_{b}$, and the total energy used for the boot-up step is $E_{b}$. The energy-saving *E* is calculated using Equation (2).

(2) $$E=\left(\left\lfloor \frac{n_{p}}{n_{s}}\right\rfloor -1\right)\;*\;\left(p_{l}t_{l}+p_{s}t_{s}\right)-\left\lfloor \frac{n_{p}}{n_{s}}\right\rfloor \;*\;\left(E_{rw}+E_{b}\right)\;(\mathrm{if}\;p_{ext}\ll p_{s};\;p_{s}\ll p_{l})$$

**Figure 7:**
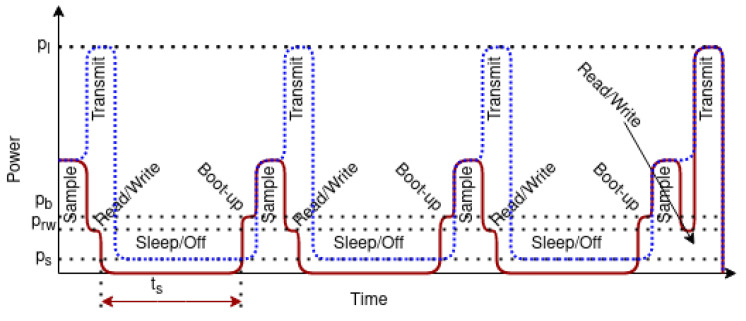
Energy savings by combining data and writing to persistent storage.

This is illustrated in Figure 7, where the blue/dotted line indicates the transmit-for-each-sample scenario and the red/solid line is the energy savings by combining data in a single data frame and writing to persistent storage. The time axis is presented to include all states but is not to scale. In reality the sleep state lasts much longer than the other events such as read-write $t_{rw}$ and boot-up $t_{b}$ ($t_{s}\gg \left[t_{rw},t_{b},t_{l}\right]$).

### 4.7. Utilizing ULP Timer

ULP timers consume electric current measured in nano-Ampere. Unlike timers that use quartz crystals, these chips have an error rate of around 1%. We proposed the use of ULP timers for IoT devices. We have measured the possibility of up to 45 times battery extension when using TPL5111 [49] chip, compared to microcontroller’s built-in ULP, for IoT nodes that are active up to 1 min per day [50]. Such nodes are primarily used to monitor environmental conditions that need infrequent measurements. The TPL5111 chip was used in combination with a DC-DC (buck) converter as a voltage regulator. The ULP timers control the power supply tough the chip enable pin on the DC-DC converter. Thus the microcontroller is completely shut off during the sleep cycle, and a separate wake-up circuit is needed to start the node before the timer expired. The TPL5111 has a DONE pin that the microcontroller can use to send a shutdown signal when it wants to start the next sleep cycle.

Ultra-low-energy timers do not use quartz crystals for measuring time due to the energy conservation goals. In the case of TPL5111 [49], the time is proportional to an external resistance. Knowing that resistance components are usually sensitive to environmental conditions, such as temperature, we can assume that such timers cannot have a precision comparable to the quartz crystal-based circuits. In many cases, we do not need such precision. For example, activating sleep monitoring sensors should be done some time in the evening and does not require second or minute precision.

Due to the imprecision of the timer, when the sensor measures a physical parameter, timestamping should be relative to the start of the microcontroller’s internal clock. When transmitting the data to the healthcare gateway, device up-time should also be sent to allow the gateway to correct the timestamp to the absolute time of the network.

While the ESP32 microprocessor has multiple low-energy states offering a balance between available resources and power efficiency, the expected energy efficiency is often not fully utilized by the microcontroller manufacturer. Not all peripherals take advantage of all the available power management. In reality, microcontrollers come with peripherals that either do not have low-power states or do not fully take advantage of those states when the microprocessor cannot use them. The most obvious example of energy waste is voltage regulators that rely on heat dissipation to provide the required voltage. Even when the microprocessor uses ultra-low power, the voltage regulator could waste energy orders of magnitude greater. This is evident from our experimental measurements shown in Table 1, conducted on Helteh’s Wireless Stick microcontroller. When the wireless stick has entered low-power mode, when the power is provided using switching converter on 3.3 V, the native voltage of the electronic components, with the input voltage to the converter of 5.00 V, the current is 2.27 mA, and the power consumption is 11.35 mW. However, when the device is powered using 5 V via the 5 V pin or the USB power, the power consumption is much higher, 81.04 mW and 65.13 mW respectively. The lowest current we were able to measure on the Wireless Stick was 2.27 mA; however, the expected current, if we take only the microprocessor ESP32, on which this microcontroller is based, in the hibernation mode, is 2.5 μA [36].

Let *C* is the battery capacity, $I_{ON}$ is the average current used during the active state of the microcontroller, and $I_{ULP}$ is the current consumed in sleep state, and *x* is the number of minutes in a day that the device is active. The battery lifetime in days *T* is expressed in Equation (3). We add the 24 h in the denominator to obtain a result in days. As the collection is infrequent and the data packet transmitted via LoRa is small, we expect most applications to have a very low duty cycle of under ten minutes per day.

(3) $$T=\frac{(C)mAh}{24h\;\cdot \;(I_{ON}\frac{x}{1440}+I_{ULP}\frac{1440-x}{1440})mA}$$

**Figure 8:**
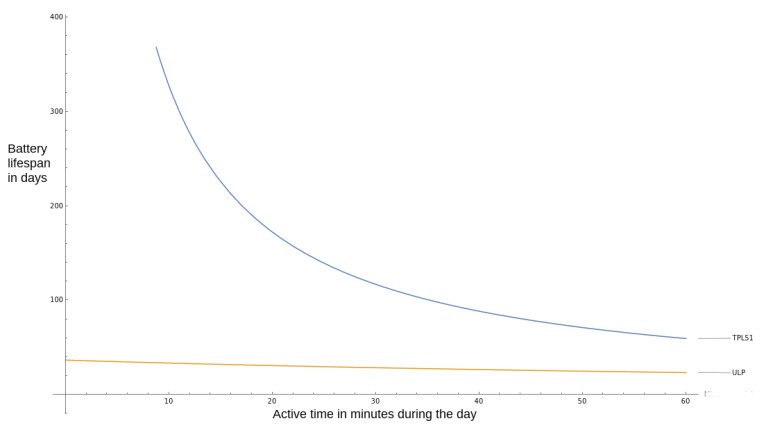
2000 mAh battery lifetime when using TPL5111 and ULP in sleep mode, based on node active period.

If we take for example a 2000 mAh battery, and we replace the measured values in Equation (3), the resulting is shown in Equation (4). We can then plot this result as shown in Figure 8, where we draw both the ratio for battery life extension using TPL5111 and the built-in ULP state of the microcontroller.

(4) $$T_{TPL5111}=\frac{2000mAh}{24h\;\cdot \;(I_{ON}\frac{x}{1440}+0.026\frac{1440-x}{1440})mA}$$

From Figure 8, we notice that sensors that operate for 10 min per day can last one year on a charge of 2000 mAh battery when using an external ULP timer. While this is the best-case scenario for this microcontroller, if we account for radio communication and other energy-intensive tasks, the energy consumption will increase.

**Figure 9:**
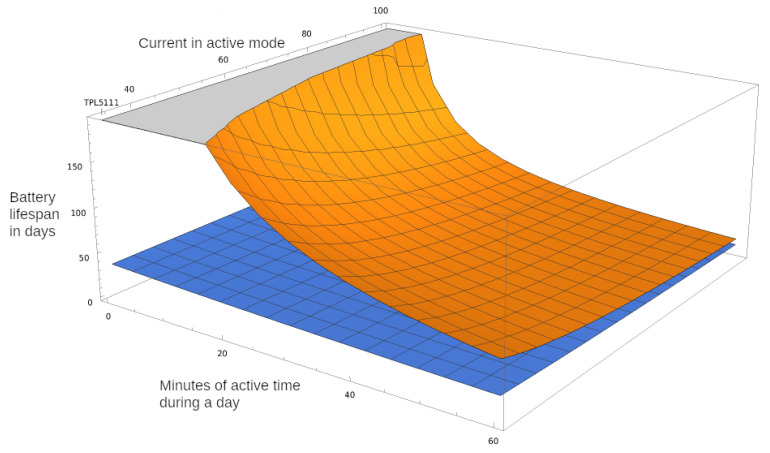
2000 mAh battery lifetime depending on active time and active average current $I_{ON}$.

In Figure 9 we also include the variable of average current during the active period from the minimal value od 33 mA to 100 mA graphed on the y-axes, while the z-axes indicate the number of days the battery charge is expected to last. The top chart in orange indicates the battery lifespan when the TPL5111 timer is used, while the bottom chart in blue indicates the usage of the onboard sleep state. The maximum shown in the plot is 200 days, and the area in gray indicates a lifespan of at least 200 days. Thus, we see that the ratio drastically increases when the active state is short and energy-efficient. For sensors that require a longer active state, the energy efficiency should further be improved by using microcontrollers capable of using the multiple ESP32 power modes [36].

## 5. Remote and Environment Triggering

The low power constraints of the architecture result in information pulling as means for the battery-powered devices to communicate in the network. This is sufficient to complete some goals, such as scheduled communication and sending data when needed. However, push notifications are necessary for specific situations, such as when a command is sent from the care providers. To allow for push notifications, we propose utilizing wake-up signals to trigger the devices to exit the sleep state and prepare to receive network communication.

In an ideal case, a sensor IoT module will not consume any energy until the act of measurement. When measuring parameters on time intervals, this can be accomplished by ULP timers. However, when monitoring for an event that might happen at any time, we either have to conduct measurement the entire time or construct a device that will wake the node upon such event occurs.

A care receiver might want to obtain sensor information at a specific time. For example, they might want to check if the person is in bed, if there is electricity in the house, or if the medicine disposal device is empty. A remote trigger mechanism is needed to send a wake-up signal to those sensors that are identified as needing these ad-hoc probes. Such a signal must be routed through the communication gateway for the system. The standby energy usage must be low enough not to affect the devices’ expected battery life significantly.

When utilizing the external switch to cut the power supply to the microcontroller, we need a way to send a wake-up signal to start the microcontroller. Fortunately, the ULP timer can receive interruptions. A wake-up signal can be used to trigger this interrupt. To “wake up” the TPPL5111, the DELAY/M_DRV pin should be set to high, which can be accomplished by a short connection to $V_{DD}$ pin.

In this paper, we consider the following types of wake up signals:

- Radio frequency (RF) signal;
- Light signal;
- Sound signal;
- Mechanical signal.

### 5.1. RF Wake-Up

The primary mode for data communication in LoRaWAN is to send data while avoiding collision and then pulling from the gateway. While it is possible to set up LoRaWAN always to receive packets from the gateway, such a setup would have energy consumption implications. Furthermore, it would prevent the deep sleep states we want to achieve. Instead, we can implement a low standby energy radio receiver as an interrupt for waking up the microcontroller and activating LoRaWAN to receive the intended packet.

Developments in CMOS power consumption have led to the birth of a new design paradigm to reduce power consumption and, in combination with energy harvesting, reach the goal of the perpetual operation [51]. This energy-saving mechanism is known as the wake-up radio (WuRx). WuRx is a very low-power secondary radio that allows the main radio to remain powered off. As soon as some information should be transmitted, the WuRx receives a wake-up signal from the device initiating the communication and then decides to switch on the main radio receiver [52]. As a result, WuRx consumes power orders of magnitude lower than the radio hardware utilized by the wireless sensor [28]. A comprehensive research review of the progress in WuRx, both for hardware and software aspects, is presented in [51].

On power management, three techniques are used: always-ON, duty cycling the WuR, or energy harvesting [51]. As the name implies, the always-ON is in a perpetual listening state, and then the wake-up signal can be detected at any time, thus minimizing latency. In contrast, the duty cycle mode periodically activates the radio receiver, requiring the wake-up signal to be at least active for the receiver’s entire power cycle duration. When the communication is sporadic, this method is inefficient for energy consumption and latency, and it cannot be used for critical applications that require high-reliability [52]. The low duty cycle approach also suffers from network latency and the need for complex protocols to achieve synchronization while power is wasted during periods of no transmission [53]. To reach the ideal case of zero DC power consumption in standby mode, the passive wake-up receivers have been proposed [54]. Passive wake-up radios are those which are using the wake-up signal itself as the energy source [52]. They are also known as energy harvesting receivers because the wake-up signal is also powering the receiver, usually by charging a capacitor sufficient time to react to the signal. Prevention of false wake-ups and maximizing signal sensitivity while consuming minimal power are challenges with implementing an always online wake-up circuit [53].

The main challenge with WuRx is device selection. As the circuit of the WuRx must be simple to ensure low energy usage, we cannot implement a real addressing scheme. When the frequency of remote triggers is infrequent, the most straightforward approach is to accept that all devices with this capability will wake up and start listening to LoRa messages. Once they determine they are not the intended target, they power off. Some techniques implement identifier schemes using a frequency spectrum pattern [54]. In [54] an identification mechanism, using frequency fingerprints, is proposed and studied. This paper presents orthogonal frequency-division multiplexing (OFDM) transmitter which can generate an identifier without any additional hardware. When the identifier is sent as a frequency spectrum pattern, a simple analog circuit may be used for device selection [52].

A WuRx has direct implications in our first user story. A WuRx can be used to activate an otherwise dormant GPS device with LoRa communication.

### 5.2. Light Wake-Up Signal

Light can be used as a wake-up signal to detect a change in the environment or as a type of remote control device to device. However, the latter has the line of sight limitation and can be used only by IoT nodes in the same room. Due to this limitation and lack of clear advantages over RF wake-up approaches such as Bluetooth, we do not consider this approach. On the other hand, sensing the light in the environment can indicate events that the IoT device should monitor. An example of such an event is turning on the light in the bedroom. Here, the challenge is to ensure that the wake-up signal is not generated during daylight. One solution is to limit the incoming light’s direction towards the light fixture and away from the windows. There are several light sensor types: photo-emissive cells, photo-conductive cells, photovoltaic cells, and photo-junction devices.

### 5.3. Sound Wake-Up

Sound wake-up detector relies on detecting sound waves and can be based on frequency detection [55]. We consider two scenarios for sound wake-up: trigger from the person or the environment and device to device trigger.

The first scenario is helpful for the user story in Section 2.3. A sound wake-up can be used to react to environmental changes. Loud noise can indicate an accident so that a sound amplitude trigger can wake up a specific sensor. In the first scenario, a patient could use voice to activate an IoT device to control the environment or provide feedback to the care providers, including calling for help. The suitable candidate for this is the piezoelectric device as described in [56]. It will only activate upon reaching the sound threshold and then process the sound. This microphone has sleep mode with a power consumption of only 6 μW. While the wake-up signal is lost after the wake-up circuit is activated [55], the sound generated during an accident is one of the most valuable pieces of information. Losing this sound limits the system’s ability to identify the event. However, the device described in [56] can go from sleep mode to full power in 200 μs, thus allowing fast reaction time to record and process sound, including a call for help.

In the second scenario, a sound beyond adult people’s hearing abilities, an ultrasound, can be used for the wake-up signal between IoT devices or between a healthcare gateway and the IoT devices. In this application, the devices do not need a line of sight. Instead, they require proximity for the sound signal to travel from the source and be detected by the target device. However, the solution presented in [55], which meets the power requirements, is too complex and costly to be implemented in the simple sensor modules.

### 5.4. Mechanical Wake-Up

A mechanical wake-up circuit is the most straightforward way to trigger a wake-up. The mechanical sensors can detect vibration, movement, tilt, bending, pressure, skin contact. Most of these sensors consume zero power when on stand-by since their mechanism is usually contact that closes an open circuit. For example, a pressure sensor placed under the mattress, a vibration sensor placed on the bed, or a modified push-down button could trigger the bed movement sensor. Similar sensors can be placed on doorknobs, door frames, or floor mats to detect when a person is moving in and out of a room or the house, which will indicate when the IoT nodes should enter or wake up from deep sleep.

We will briefly describe several of these mechanical switches.

- Reed switch has two contacts that are very close to each other but normally separated. In the presence of a magnet, they will touch and close the circuit.
- Tilt switch uses a metal ball or a drop of mercury that, when tilted, drops toward two contact leads and makes a closed circuit between them.
- Vibration sensor has a sturdy metal wire in the middle surrounded by an elastic metal spring. The spring does not touch the wire even when titled but will touch if there is a vibration.
- Bi-metal thermostat detects temperature changes. Depending on the model, it makes contact closing the circuit for temperatures above or below the set value.
- Pressure switch/button creates contact on pressure.

**Figure 10:**
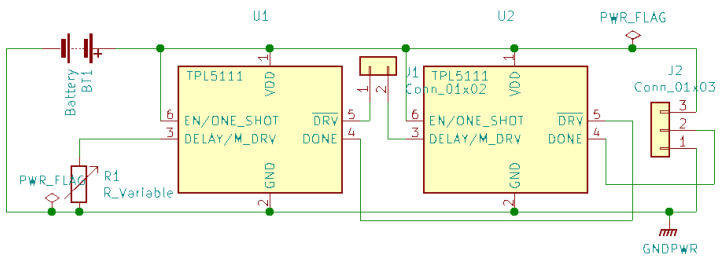
Mechanical wake-up with delay circuit.

The main challenge with this kind of sensor is a frequent wake-up signal. For example, a vibration or pressure sensor can be placed in the person’s shoes to activate when they are wearing them. However, this would mean a wake-up signal is sent with each step. The workaround is to have a secondary ultra low power timer (i.e., TPL5111) that would be placed before the mechanical switch, as shown in Figure 10.

## 6. Discussion

The increased life-expectancy of people due to improved living standards would burden medical systems. Connected healthcare provides an opportunity for affordable and sustainable medical services. The current research takes advantage of well-established network infrastructure and thus offloads the communication component to prior investments. Enhanced living environments (ELE) and smart homes could establish a common framework for providing services and interaction with inhabitants of the house [57]. However, a healthcare system for the rural population would not benefit or would have limited benefits from such infrastructures. For this reason, a new architecture focused on the constraints associated with rural environments is needed.

We review several diverse scenarios that present a starting point to identify challenges the system would face to address this issue. These scenarios should be analyzed in the context of a rural environment. We can illustrate this point for the lost person scenario. In urban areas, such cases would be reported to emergency services that have resources that, when mobilized, could extend the search in a large geographical area. Eyewitnesses and social media could be used to locate the person. Technology assistance is also available, including phone tracking of internet-connected devices, cell tower-based locations. Bluetooth tags attached to clothes or personal items could also be used [58]. Each of these options is very limited or nonexistent in rural areas. Emergency services are usually located at a greater distance and have fewer staff and resources. There is less chance of finding eyewitnesses, and using technology to locate a person is very limited. The case of environmental and safety monitoring could be considered a part of the smart home system. Designing or upgrading houses for smart homes is more difficult for rural places, considering that some solutions require cloud connection, and the investment could be prohibitively expensive.

The main challenges in implementing the connected healthcare system in rural areas arise from the high likelihood of lack of connectivity infrastructure, including mobile networks, and the need to make the system affordable. A viable system should be self-sufficient and assume the worst case, rather than the best case, from the point of view of established infrastructure. Thus, we assume no prior network exists for the system to take advantage of. We analyzed the existing network solutions, including extended-range networks, and we identified that the LPWAN network is the best fit. The main factor in determining this is to ensure the minimal cost for installing few IoT devices to address a specific need instead of requiring a full deployment for each household. In the extreme case, we would have a single IoT device deployed, in which case the device should have a communication link to the network without deploying additional network devices. In addition, all IoT devices are required to have a battery operation capability as, in some cases, external power might not be available or require additional installation.

Based on the previous comparison of LPWAN technologies [5], we are using LoRa communication with the LoRaWAN MAC layer for network communication. We propose a fog-based architecture emphasizing offloading data collection, pre-processing, and partial decision-making to the e-healthcare gateways. Due to the limited bandwidth of LoRa and the need for energy preservation, each node in the fog network should maximize longevity unless a priority event is suspected that requires the full performance of the system. Long-term data storage exists at each network layer to minimize bandwidth utilization while preserving valuable data. This data is extracted during servicing of the devices.

In addition to reducing network utilization, there are additional tactics that can be used for energy conservation. We found that the most commonly addressed approach is minimizing energy usage by tweaking the LoRa parameters. We identify and provide scenarios for other means to reduce energy usage, focusing on the device’s smart duty cycle. While modern microcontrollers, such as the ESP-32, can have multiple sleep stages, nano-Ampere timers can be used to push this approach even further. The energy consumption of the sensors must also be minimized by a balanced approach of reducing the duty cycle of the sensors while still collecting the data when needed. Variable scanning resolution is used to ensure valuable data is collected and wasteful cycles are minimized.

The battery extension that we have achieved is in the range of tens of times, depending on the utilization profile of the microcontroller, for low utilization even up to 80 times longer battery life [50]. In [59], the authors have achieved 95.2% reduction, and they measured a sleep mode current of 33 nA. While we have achieved 99% reduction, this is due to the higher current that our chosen microcontroller draws during sleep. A similar decrease in inactive current by 90–95% was shown in [60]. In [61], the authors have achieved 40 times battery extension using an event-driven system for continuous and long-term operation of a wireless sensor to monitor civil infrastructure given a 1% event probability. While direct comparison cannot be measured due to the difference in the power profile of the IoT microcontroller and the implementation, our results are comparable to the ones published in the literature.

While the proposed architecture allows rural healthcare to be implemented, the constraints of low network bandwidth and the need to reduce the active time of the IoT nodes limit the capabilities of the system. For example, sensors that generate a large quantity of data cannot effectively be used in the proposed architecture. In addition, the proposed architecture is very sensitive to bugs in firmware and software that prevent the device from booting correctly or entering a low power state. Such bugs could drain the battery rendering the node unusable until it is serviced, decreasing the benefits of the proposed architecture.

We expect the most challenging aspect of the deployment of the proposed architecture to be the initial deployment, specially the communication hub that would require power supply and battery backup to ensure reliable operation.

## 7. Conclusions and Future Work

The rural population often lacks access to quality healthcare. This issue is not new, and often the urban areas have more staff and better equipped medical institutions. Connected healthcare is a paradigm that is promising to reduce healthcare costs and provide better access. However, research is often focused on taking advantage of the latest developments in communication networks, including 5G technologies. In contrast, the rural population is left behind on this promise for better, more affordable healthcare. We describe several user stories in order to illustrate the constraints that are imposed in low connectivity areas. We then design architecture that would work within the given constraints. The architecture considers the sparse distribution of houses in rural communities, the lack of infrastructure for smart homes, and the high cost of converting homes. Thus the IoT sensors are assumed to be battery-powered, and the service interval is infrequent. We proposed multiple techniques for energy conservation, and we conducted measurements showing that external ULP timers can be used to extend battery life in the order of tens of times, depending on the usage profile of the IoT node. While ULP deep sleep states can extend the battery life for years, the system’s usability is evaluated by being responsive when needed. For this, we propose wake-up triggers for the microcontrollers and the sensors. Certain observed events could act as triggers either by generating an electrical charge or closing an open circuit, thus allowing for zero energy sensors. Sensors that detect mechanical changes such as pressure contacts detect and also trigger an event. Very low-power wake-up signals are used for resuming the operation of a microcontroller in a sleep state. These triggers include RF wake-up and light and sound wake signals.

In the future, we will create granular energy profiles of the microcontrollers and sensors for each operational stage. Then, using the architecture presented in this paper, we can design a specific usage profile that would minimize energy consumption for given functional expectations of the IoT nodes. Having this data, we can simulate the battery usage of the IoT nodes and develop battery specifications for each type of sensor device.

In addition, we will investigate how different ML algorithms and approaches perform in the proposed architecture, especially given the network constraints, and influence its main benefits.

## Figures and Tables

**Table 1:** Power consumption based on Vdd input pin [50].

State	Voltage	Current	Power
Using micro USB connector			
Wireless Stick ON	5.01 V	51 mA	255.51 mW
Wireless Stick low power	5.01 V	13 mA	65.13 mW
Using 5 V pin			
Wireless Stick ON	7.01 V	44.45 mA	309.49 mW
Wireless Stick low power	7.09 V	11.43 mA	81.04 mW
Power OFF by TPL5111	7.11 V	37 μA	0.26 mW
Using 3.3 V pin			
Wireless Stick ON	4.91 V	32.95 mA	161.78 mW
Wireless Stick low power	5.00 V	2.27 mA	11.35 mW
Power OFF by TPL5111	5.00 V	26 μA	0.13 mW

## Data Availability

Not applicable.
